# Human placenta-derived neurospheres are susceptible to transformation after extensive *in vitro* expansion

**DOI:** 10.1186/scrt444

**Published:** 2014-04-22

**Authors:** Donatella Amendola, Marta Nardella, Loredana Guglielmi, Lidia Cerquetti, Elisabetta Carico, Viola Alesi, Manuela Porru, Carlo Leonetti, Claudia Bearzi, Roberto Rizzi, Igea D’Agnano, Antonio Stigliano, Giuseppe Novelli, Barbara Bucci

**Affiliations:** 1 Research Center San Pietro Hospital-Fatebenefratelli, Via Cassia 600, 00189 Rome, Italy; 2 CNR-Institute of Cell Biology and Neurobiology, Rome, Italy; 3 Endocrinology Department of Clinical and Molecular Medicine, Sant’Andrea Hospital, Faculty of Medicine and Psychology, Rome, Italy; 4 Cytopathology Department of Clinical and Molecular Medicine, Sant’Andrea Hospital, Faculty of Medicine and Psychology, Rome, Italy; 5 Medical Genetics Unit San Pietro Hospital Fatebenefratelli, Rome, Italy; 6 Experimental Chemotherapy Laboratory, Regina Elena National Cancer Institute, Rome, Italy; 7 CNR-Institute of Cell Biology and Neurobiology, Rome; I.R.C.C.S. Multimedica, Scientific and Technology Pole, Milan, Italy

## Abstract

**Introduction:**

The cancer stem cell model links neoplastic cells with normal stem cell biology, but little is known on how normal stem cells are transformed into cancer stem cells.

**Methods:**

To investigate the processes underlying the transformation of normal stem cells we developed *in vitro* a cancer stem cell model from human amniotic and chorionic placenta membranes. In this model we studied the expression of specific stem cell molecules by flow cytometry, and genes, by real time RT-PCR. Microscopy immunfluorescence was employed to investigate the proliferative and differentiation patterns. Fluorescence microscopy and FACS were employed to investigate the proliferative and differentiation patterns. To evaluate the tumorigenic potential of our model we injected the cells into NOD.CB17-Prkdc^scid^/NCrHsd mice.

**Results:**

Normal human stem cells from amniotic and chorionic placenta membranes were converted into neural cell lineages, under specific conditions, to form secondary neurospheres with a capacity for self-renewal. After extensive *in vitro* culture, these cells underwent spontaneous transformations and acquired a neuroblastoma (NB)-like phenotype with an elevated proliferative potential that is comparable to established neuroblastoma cell lines. The ability of these cells to transform their phenotype was evidenced by increased clonogenic ability *in vitro;* by augmented expression level of certain proliferation- and transformation-related genes (e.g., *CCNA2*, *MYCN*, *ENPP2*, *GRIA3*, and *KIT*); by the presence of multinucleated and hyperdiploid cells. We further demonstrated that the transformed phenotype is an NB by measuring the expression of NB-specific markers, disialoganglioside GD2 and N-Myc proteins.

**Conclusions:**

We have developed a cancer stem cell model starting from normal human stem cells derived from amniotic and chorionic placenta membranes. These cells are able to differentiate into neural cell lineages and to undergo spontaneous transformations and acquire an NB-like phenotype.

## Introduction

Neuroblastoma (NB) is a very aggressive solid tumour and is the most commonly diagnosed solid tumour in children [1,2]. Although NB disease remission can frequently be achieved for patients *via* a combination of surgery, radiation and chemotherapy, relapse is very common. Recent studies demonstrate that NB is generated and maintained by a small cell population of undifferentiated cells (1% to 2% of the total), which are identified as the tumour-initiating cells (TICs) and are commonly defined as cancer stem cells (CSCs). These cells play an important role in carcinogenesis and tumour progression [3]. There is increasing evidence confirming the presence of CSCs in other solid tumours, including breast, brain, prostate, colon and lung cancers, as well as haematopoietic tumours, such as leukaemia [4-9]. These cells are characterised by extensive potential for self-renewal (serial sphere formation) driving tumourigenesis [10]. They show a multi-drug resistance phenotype and express prominin 1 (CD133), a surface marker of normal stem cells [3,11,12]. Tumour tissue-derived CSCs are usually used as a model to study the biological properties of CSCs in solid tumours [3,13,14]. However, because CSCs represent a very small subset of tumour cells, the molecular mechanisms involved in expansion and neoplastic transformations have yet to be elucidated. Therefore, more insight into the molecular mechanisms that predispose normal stem cells to undergo malignant transformations is needed and may help develop selective therapeutic strategies to target CSCs. To study the formation of CSCs, different models derived from normal adult or embryonic tissues, which were spontaneously or forcedly transformed, have been developed. Gro Vatne Røsland and colleagues characterised a model of human adult mesenchymal stem cells (MSCs) derived from normal [15] bone marrow that undergo spontaneous malignant transformation following *in vitro* culture.

Milyavsky and collaborators [16] reported that a prolonged culture of telomerase-immortalised human fibroblasts also acquired a pre-malignant phenotype. In addition, Okamoto and colleagues [17] provided a genomic characterisation of CD133-positive stem cells derived from umbilical cord blood and stimulated the cells to proliferate (*in vitro* expansion) with estradiol; in this study, they identified genes and signalling pathways involved in both stem cell expansion and haematological cancer development [17]. Although the use of embryonic tissues after long-term culture expansion appears to be advantageous in terms of expansion potential and susceptibility to malignant transformation compared with adult tissues, ethical issues limit the use of these tissues.

In this paper, we demonstrate that human placental foetal tissues (amnion and chorion membranes) maintaining most of the embryonic properties could represent a physiologic pluripotent model of MSCs not obtained by forced genetic reprogramming of somatic cells. We also converted MSCs into neural lineages by spheres forming under specific conditions, and after extensive culture *in vitro* adherent placenta-derived (PD) neurospheres undergo spontaneous transformations and acquire an NB-like phenotype. It is noteworthy that placental tissues are normally discarded after birth, abundantly available and ethically unobjectionable, thus overcoming the ethical concerns related to the use of umbilical cord blood [2].

## Methods

### Ethics statement

The study was approved by the Ethics Committee of San Pietro Hospital Fatebenefratelli (64/2012/cb) and all participants gave written, informed consent.

The procedures involving mice and their care were in compliance with Regina Elena National Cancer Institute animal care guidelines and with international directives (directive 2010/63/EU of the European parliament and of the council; Guide for the Care and Use of Laboratory Animals, United States National Research Council, 2011).

### Isolation and culture of MSCs from human placentas

The human term placentas were collected from a cohort of 35 women at gestational weeks 39 ± 1. Mean maternal age was 30 years. Following informed consent, placentas were collected immediately after elective caesarean section in the absence of labour, preterm rupture of membrane, chorioamnionitis or chromosomal abnormalities. From each placenta, we collected the foetal membranes (amnion and chorion) and washed them in physiologic saline solution. We proceeded to do a mechanical digestion and then, the tissue was subjected to three sequential enzymatic digests with trypsin and deoxyribonuclease I (DNase I) (Invitrogen, Carlsbad, CA, USA). After each enzymatic digestion, cell suspensions were filtered through a 100 μm cell strainer and finally, the cells were collected by centrifugation for five minutes at 200 × *g*. In order to expand MSCs, the cell pellets were suspended and cultured at 37°C in a 5% CO_2_/95% air atmosphere in (Dulbecco’s) modified Eagle’s medium ((D)MEM) supplemented with 20% foetal calf serum (FCS), 2% penicillin/streptomycin, 1% L-glutamine and 2% Fungizone Amphotericin (Gibco, Grand Island, NY, USA). Cells grew as fibroblastic cells that developed into visible colonies after seven days of culture and after twenty days reached 90% confluence. At this time, we recovered the cells using a trypsin- ethylenediaminetetraacetic acid (EDTA) solution for culture expansion and we expanded the cells until 29 population doublings (pd, corresponding to passage 20 and calculated as described below). Every 6 pd (corresponding to 4 passages) PD-MSCs were characterised for cell growth, phenotypic profiles and gene expression up to 29 pd.

### Cell growth

For the cell growth curve, cells were seeded in six-well culture plates at a density of 5 × 10^4^ cells/well, cultured at 37°C and 5% CO_2,_ and viable cells were counted (trypan blue dye exclusion test) every day from day 1 to day 12 of culture. The pd of cultured PD-MSCs and adherent PD neurospheres expanded was calculated at every passage according to the equations log_10_ (the number of harvested cells/the number of seeded cells) and log_2_ (the number of harvested cells/the number of seeded cells), respectively. The finite pd were determined by cumulative addition of total numbers generated from each passage considered.

### Characterisation of PD-MSCs by flow cytometry

Cultured cells were harvested and the expression of cell surface markers was analysed by direct or indirect immunofluorescence using a FACSCalibur cytofluorimeter (Becton Dickinson, Sunnyvale, CA, USA). For direct immunofluorescence, the PD cells were incubated with fluorescein isothiocyanate (FITC)-conjugated antibodies against human highly polymorphic class I leukocyte antigens (HLA-ABC) (Becton Dickinson); human class II leukocyte antigen (HLA-DR) of the major histocompatibility complex (MCH) (BD-Pharmingen, San Diego, CA, USA); different cluster of differentiation (CD), such as CD44 molecule Indian blood group (CD44) (Miltenyi Biotec, Calderara di Reno, Italy) or Thy-1 cell surface antigen (CD90) (Miltenyi Biotec); or with phycoerythrin (PE)-conjugated antibodies against protein tyrosine phosphatase, receptor type C (CD45) (Miltenyi Biotec), CD133 (Miltenyi Biotec) and endoglin (CD105) (Miltenyi Biotec) for one hour on ice and immediately analysed by flow cytometry (FCM). For indirect immunofluorescence, cells were incubated with the primary antibodies 5'-nucleotidase, ecto (CD73) (Santa Cruz Biotechnology, San Diego, CA, USA), integrin beta 1 fibronectin receptor, beta polypeptide, antigen (CD 29) (Stemgent, San Diego, CA, USA), stage-specific embryonic antigen-4 (SSEA-4) (Santa Cruz Biotechnology), UDP-Gal:betaGlcNAc beta 1,3-galactosyltransferase, polypeptide 5 (SSEA-3) (Santa Cruz Biotechnology) or octamer-binding transcription factor 4 (Oct-3/4) (Santa Cruz Biotechnology) for one hour on ice and then incubated with a secondary FITC-conjugated antibody for 50 minutes on ice and immediately analysed by FCM.

### Induced pluripotent stem (iPS) cells

Lentiviral vectors employed for the induction of reprogramming harbored bicistronic plasmids expressing *Oct-3/4* and either *Sox2* or *Klf4* (*OSK*). The original vector was kindly provided by Prof. Luigi Naldini (Fondazione San Raffaele, Milan, Italy). Low passage 293 T cells (Cell Biolabs, San Diego, CA, USA) were used to produce lentiviruses expressing each pair of transgenes, using the pPAX2 and pVSV-G packaging constructs and a calcium phosphate transfection protocol. Supernatants containing OSK lentiviruses were collected 48 hours later, filtered and used freshly right after preparation. To generate iPS, human adult skin fibroblasts were exposed to a mixture of equal volumes of the two OSK lentiviral vectors. Four days after transduction, cells were trypsinised and plated on a mouse MEF-feeder layer (Millipore, Billerica, MA, USA) and cultured in propagation medium composed of knock-out (D)MEM containing 20% knock-out serum replacement, L-glutamine, non-essential amino acids, basic fibroblast growth factor (bFGF), B27, N2 supplement, β-mercaptoethanol, penicillin (100 U/ml) and streptomycin (100 mg/ml). Expression of embryonic genes was analysed by qRT-PCR. Total RNA was extracted from cells using Trizol (Invitrogen). A quantity of 1 μg of total RNA was reverse transcribed to cDNA using SuperScript III Kit (Invitrogen) to be amplified by qRT-PCR. Sybr Green PCR master mix (Applied Biosystems, Barlington, ON, Canada) and primers specific for markers of pluripotency were used. Samples were analysed in triplicate using the Tetrad 2 (Biorad, Richmond, CA, USA) and 7900HT qRT-PCR (Applied Biosystems). Immunofluorescence staining was performed on cells cultured on petri dishes, fixed in 4% paraformaldehyde and permeabilised with 0.5% Tween 20. The cells were then incubated with the primary antibodies, such as Oct-3/4 (Abcam, San Francisco, CA, USA), Nanog (Abcam), SSEA4 (Stemgent). To verify teratoma formation, 5 × 10^5^ human iPS cells were injected subcutaneously in five NOD^scid^ mice. Tumours were sought after four weeks. iPS cells were used as control cells for the analysis of stemness genes.

### Western blot analysis

Samples were solubilised in urea buffer on ice. Protein content in the different samples was quantified by using the Bradford method (Bio-Rad). Aliquots (25 μg) of proteins were subjected to 10% sodium dodecyl sulphate-polyacrylamide gel electrophoresis (SDS-PAGE) and the resolved proteins were blotted on a nitrocellulose membrane, which was then blocked in TBS buffer (20 mM tris Base, 137 mM NaCl, 1 M hydrochloric acid, pH 7.6) containing 5% nonfat dry milk (Bio-Rad) for at least one hour. Blots were then incubated with the primary antibodies anti-Oct-3/4 (Becton Dickinson) and anti-β-actin (Sigma, San Louis, MO, USA). Peroxidase-labelled anti-mouse immunoglobulin G (IgG) (Sigma) was used as secondary antibody. Immunoblots were processed for enhanced chemiluminescence detection (Amersham Life Sciences, Little Chalfont, Buckinghamshire, UK). The relative amount of transferred proteins in a given sample was quantified by scanning X-ray films and by densitometry analysis (Total Lab image analysis solution, version 2003, Nonlinear Dynamics, Newcastel upon Tyne, UK).

### Total RNA preparation

Total RNA was isolated from each sample of uncultured placenta, PD-MSCs, secondary PD-neurospheres and expanded PD-neurospheres using a Total RNA Purification Kit (Norgen Biotek Corporation, Thorold, CA, USA) according to the manufacturer’s instructions. The RNA quantity was determined by absorbance at 260 nm using a NanoDrop UV–VIS spectrophotometer. The quality of each sample was checked with an Agilent BioAnalyzer 2100 (Agilent RNA 6000 Nanokit). Samples with an RNA integrity number (RIN) lower than 8.0 were discarded.

### Real-time reverse transcriptase polymerase chain reaction (RT-PCR) analysis

RNA (500 ng) was retro-transcribed according to standard conditions and the cDNA was then subjected to real time PCR analysis with an Applied Biosystems 7900HT thermal cycler, using the SensiMixSYBR Kit (Bioline, London, UK) and the following specific primers: nanog homeobox, *hNANOG* (sense (s): AGATGCCTCACACGGAGACT, antisense (as): TTTGCGACACTCTTCTCTGC); prominin 1, *hPROM1* (s: TCCACAGAAATTTACCTACATTGG, as: CAGCAGAGAGCAGATGACCA); SRY sex determining region Y-box 2, *hSOX2* (s: TGCTGCCTCTTTAAGACTAGGAC, as: CCTGGGGCTCAAACTTCTCT); SRY sex determining region Y-box 9, *hSOX9* (s: GTACCCGCACTTGCACAAC, as: TCGCTCTCGTTCAGAAGTCTC); POU class 5 homeobox 1, *hPOU5F* (s: CTTTGAGGCTCTGCAGCTTAG, as: GGTTTCTGCTTTGCATATCTCC); polycomb ring finger oncogene, *hBMI1* (s: TTCTTTGACCAGAACAGATTGG, as: GCATCACAGTCATTGCTGCT); ATP-binding cassette, sub-family G (WHITE), member 2, *hABCG2* (s: TGGCTTAGACTCAAGCACAGC, as: TCGTCCCTGCTTAGACATCC); cyclin A2, *hCCNA2* (s: GGTACTGAAGTCCGGGAACC as: GAAGATCCTTAAGGGGTGCAA); cell e-box transactivator 2, *hNEUROD1* (s: CTGCTCAGGACCTACTAACAACAA, as: GTCCAGCTTGGAGGACCTT); nestin, *hNES* (s: TGCGGGCTACTGAAAAGTTC, as: TGTAGGCCCTGTTTCTCCTG); vimentin, *hVIM* (s: GTTTCCCCTAAACCGCTAGG, as: AGCGAGAGTGGCAGAGGA); myelocytomatosis viral related oncogene neuroblastoma derived, *hMYCN* (s: CCACAAGGCCCTCAGTACC, as: TCCTCTTCATCATCTTCATCATCT); v-kit Hardy-Zuckerman 4 feline sarcoma viral oncogene homolog, *hKIT* (s: CGTGGAAAAGAGAAAACAGTCA, as: CACCGTGATGCCAGCTATTA); ectonucleotide pyrophosphatase/phosphodiesterase 2, *hENPP2* (s: GCACATCGAATTAAGAGAGCAG, as: GGGGGAGTCTGATAGCACTG); glutamate receptor ionotropic AMPA3, *hGRIA3* (s: CCTCTATGACACAGAACGAGGA, as: TGCACTGCTGCTTCCATAAT); TATA box binding protein, *hTBP* (s: GAACATCATGGATCAGAACAACA, as: ATAGGGATTCCGGGAGTCAT); glyceraldehyde-3-phosphate dehydrogenase, *hGAPDH* (s: AGCCACATCGCTCAGACA, as: GCCCAATACGACCAAATCC); protein phosphatase 1, *hPPIA* (s: ATGCTGGACCCAACACAAAT, as: TCTTTCACTTTGCCAAACACC); CDKN2A**,***hCDKN2A* (s: TGCCTTTTCACTGTGTTGGA, as: TGCTTGTCATGAAGTCGACAG).

### Conversion of PD-MSCs into spheres neural cells

PD-MSCs from 6 pd (corresponding to passage 4) were plated in nonadherent conditions: serum-free neurobasal medium (Gibco), supplemented with 20 ng/ml epidermal growth factor (EGF) (Sigma), 40 ng/ml bFGF (Sigma) and 1% neuronal supplements N2 (Gibco) and B27 (Gibco), and 2 μg/ml heparin using 60 mm low-attachment culture dishes at a density of 1.9 × 10^6^ cells/dish. After four days from seeding, the cells formed primary floating neurosphere-like structures. These structures grew rapidly until day 7. At this time, before the obtained neurosphere-like structures became necrotic, we harvested them and resuspended them in Accutase enzymatic solution (Gibco) for five minutes at 37°C and then mechanically dissociated them into a single cell suspension. The cells were re-seeded in the same non-adherent conditions as above, and the secondary spheres were allowed to form. This protocol was applied for more rounds of spheres formation. As positive or negative controls we used NB LAN-5 (kindly provided by Dr. Doriana Fruci) and SK-N-SH (purchased from American Type Culture Collection, Manassas, VA, USA) cell lines, respectively.

### Characterisation of secondary neurosphere

Secondary PD-neurospheres were dissociated as previously described. Cells were incubated with the primary antibodies directed against proliferation-related Ki67 antigen (BD-Pharmingen, Franklin Lake, NJ, USA), nestin (Santa Cruz Biotechnology), vimentin (Sigma), CD133 (Miltenyi Biotec), cleaved Notch1 (Cell Signaling, Boston, MA, USA) for one hour on ice and then with a secondary FITC-conjugated antibody for fifty minutes on ice and immediately analysed by FCM.

### Differentiation assays

For neuronal differentiation, adherent PD-neurospheres were seeded in (D)MEM supplemented with 20% FCS, 2% penicillin/streptomycin, 1% L-glutamine and 2% Fungizone Amphotericin. After 24 hours they were treated with 10 μM retinoic acid (RA) (Sigma). For morphological analysis cells were seeded in 100 mm dishes (Becton Dickinson) at a density of 2 × 10^5^ cells and after three days of RA treatment the cells were viewed with an inverted phase contrast microscope at 40X magnification. The cells were scored as differentiated if the length of the neurite extensions was at least two times the diameter of the cell body. Tracing of the individual neurites and branch points was evaluated based on the cells bearing neurites as determined from the cell culture images. A total of >300 cells was examined in five randomly chosen fields in each treated and untreated sample. The projection images were semiautomatically traced with NIH ImageJ using the NeuronJ plugin and the total dendritic length of each individual neuron was analysed.

For immunofluorescence assays cells were seeded onto multi-chamber slides (Becton Dickinson) at a density of 1 × 10^4^ cells. After one, three and seven days of treatment, the slides were washed in phosphate-buffered saline (PBS) 1X with calcium and magnesium, fixed in paraformaldehyde 4% for 15 minutes and permeabilised in Tris buffered saline (TBS) 0.05 M pH 7.4 with 1% Triton X-100 for 15 minutes. After blocking in TBS 10% serum for 30 minutes, slides were incubated with anti**-**glial fibrillary acidic protein (GFAP) (Dako, Glostrup, Denmark) antibody overnight at 4°C. After washing in TBS, they were incubated with goat anti-rabbit Alexa Fluor 488 antibody (Invitrogen) for 30 minutes and finally washed twice in TBS. For other labelling, slides were permeabilised with triton 0.1% for eight minutes, washed twice in PBS 1X and incubated with anti-neurofilament 200 (NF200) (Sigma) antibody, neuronal nuclei (NeuN) (Chemicon, Millipore, Milan, Italy) and βIII tubulin (Santa Cruz) antibodies for one hour. After three washes in PBS 1X, the slides were incubated with goat anti-mouse Alexa Fluor 594 (Invitrogen) or with goat anti-rabbit Alexa Fluor 488 (Invitrogen) antibodies for 30 minutes. Nuclei were counterstained with Hoechst 33342 (Sigma) 1 μg/ml for 10 minutes. Images were acquired with an Olympus BX51 fluorescence microscope and analysed with I.A.S. software (Delta Sistemi, Legnano, Italy). The brightness and contrast of the acquired images were adjusted, and the figures were generated using Adobe Photoshop 7.0. Negative controls, that is, cells not treated with RA, were also included.

For the membrane potential measurement, cells were seeded in 100 mm (Becton Dickinson) at a density of 2 × 10^5^ cells and after one, three and seven days of RA treatment were analysed by FCM using the membrane-potential-sensitive dye 3,3’-dipentyloxacarbocyanine iodide (DiOC_5_). DiOC_5_ was added to the cell suspensions at a final concentration of 50 nM. Valinomycin (Sigma) (final concentration 5 μM) treated samples were used as hyperpolarised controls.

### Cell cycle analysis

The cell cycle was evaluated using propidium iodide (PI) (MP Biomedicals, Solon, OH, USA) staining and FCM analysis. Adherent PD-neurospheres were harvested, fixed in 70% ethanol for at least one hour and stained with a solution containing 50 μg/ml PI and 75 KU/ml ribonuclease (RNase) (Sigma) in PBS 1X for 30 minutes at room temperature. Twenty thousand events per sample were acquired. The percentages of the cell cycle distribution were estimated on linear PI histograms by using the MODFIT software.

### BrdU incorporation

Adherent PD-neurospheres were seeded onto multi-chamber slides at a density of 2 × 10^4^ cells and were cultured for 24 hours, and then 10 μM bromodeoxyuridine (BrdU) was added to the medium for 30 minutes. Cells were fixed in a solution of acetone and methanol (1:1) for 15 minutes at -20°C. Cells were rinsed with PBS 1X for 10 minutes and incubated with 3 N chloridic acid (HCL) for 20 minutes. Cells were washed twice with borax-borate buffer (pH 9.1) to neutralise the acidic pH and three times with PBS 1X. The samples were incubated with monoclonal antibody anti-BrdU (Roche, Mannhem, Germany) in complete medium containing 0.5% Tween-20 at 4°C for one hour. After being washed twice in PBS 1X, cells were exposed to FITC-conjugated F(Ab’)_2_ anti-mouse (Dako) in PBS at 4°C for 30 minutes. Finally, the cells were washed twice with PBS 1X and nuclei were then counterstained with Hoechst 33342 (Sigma), 1 μg/ml for five minutes. Immunofluorescence was carried out using an inverted fluorescence microscope (Leica Microsystems GmbH, Wetzel, Germany). Images were acquired using Canon Remote Capture software and enhanced with Adobe Photoshop CS. As a positive control, we used the NB cell line LAN-5; we also used negative controls without the antibody.

### Colony-forming assay

Adherent PD-neurospheres were seeded at clonal density (1.5 × 10^3^ cells) in a 60-mm dish. Fifteen days after seeding, the cells were fixed for one hour with a solution of 1% Coomassie Blue R250 (Bio-Rad, Hercules, CA, USA) in ethanol. The dishes were then washed with ddH_2_O, and the colonies (at least 50 cells) were counted. The results were expressed as plating efficiency (percentage of colonies formed out of cells seeded). LAN-5 and SH-SY5Y cells (purchased from American Type Culture Collection) were used as controls.

### FCM ploidy measurement

Ploidy was measured using normal human lymphocytes as internal standard and expressed as DNA index (DI), which represents the ratio of relative G0/G1 DNA content versus normal diploid lymphocyte. Thus, a DI value of 1.0 is synonymous with a normal diploid DNA content. The cells were processed as described above for cell cycle analysis. The standard sample (lymphocytes) was prepared as a single sample or mixed with the unknown samples at a concentration of 1:2. NB LAN-5 cells were used as control for aneuploid tumour cells. A total of at least 30,000 cells was measured at a flow rate of less than 200 cells/second to minimise coincidences of fluorescence signals. The coefficient of variation of the G0/G1 peak of the samples measured ranged from 0.9% to 5.0%, for a mean of 3.0%.

### Cytological analysis

The cytology of the adherent PD-neurospheres expanded was studied by Papanicolau staining after 41 pd in culture. Uncultured PD-neurospheres and LAN-5 NB cells were used as normal or tumour control, respectively.

### Analysis of the neuroblastoma GD2 and N-Myc markers

Adherent PD-neurospheres expanded *in vitro* for different passages were analysed by indirect immunofluorescence. Cells were incubated with the antibody GD2 (Abcam, Cambridge, UK) and with N-Myc (Santa Cruz) for one hour on ice and then with a FITC-conjugated antibody for 50 minutes on ice and immediately analysed by FCM. LAN-5 NB cells were used as a positive control.

### *In vivo* experiments

NOD.CB17-Prkdc^scid^/NCrHsd male 5-week-old mice were purchased from Harlan Laboratories (San Pietro al Natisone (UD), Italy). Six mice were injected subcutaneously in the flanks with adherent PD-neurospheres transformed and expanded for 41 pd, corresponding to 19 passages **(**10^6^ cells/mouse in 200 μl of Matrigel; BD Biosciences- Discovery Labware). Mice were inspected weekly for the presence of tumour up to five months and then sacrificed for histological analysis. Biopsies were fixed in 4% formalin overnight and processed through an ethanolic dehydration series for paraffin embedding. Five micrometer paraffin sections were cut for haematoxylin and eosin staining.

To assess reactivity for human HLA-ABC antigen, immunohistochemistry was performed using a human monoclonal antibody anti-HLA class 1 ABC (Clone ab70328 Abcam, Cambridge, UK) according to the standard streptavidin-biotin peroxidase complex method. The reaction was developed by adding a diaminobenzidine-tetrahydrochloride (DAB) chromogen mixture. After haematoxylin counterstaining, slides were permanently mounted and analysed for the presence and distribution of the immunostaining.

### Statistical analysis

The data are expressed as mean values ± standard deviation (SD). Statistical significance of differences between groups was tested by paired Student’s t-test or, if there were more than two groups, by one-way analysis of variance (ANOVA).

## Results

### Isolation and characterisation of PD-MSCs

We isolated and analysed cells obtained from amnion and chorion full-term placental membranes. A selection was performed using the classic adhesion method on culture plastic. The protocol was successful in 30 of the 35 placentas collected. We show the results relative to three representative placentas.

**Figure 1:**
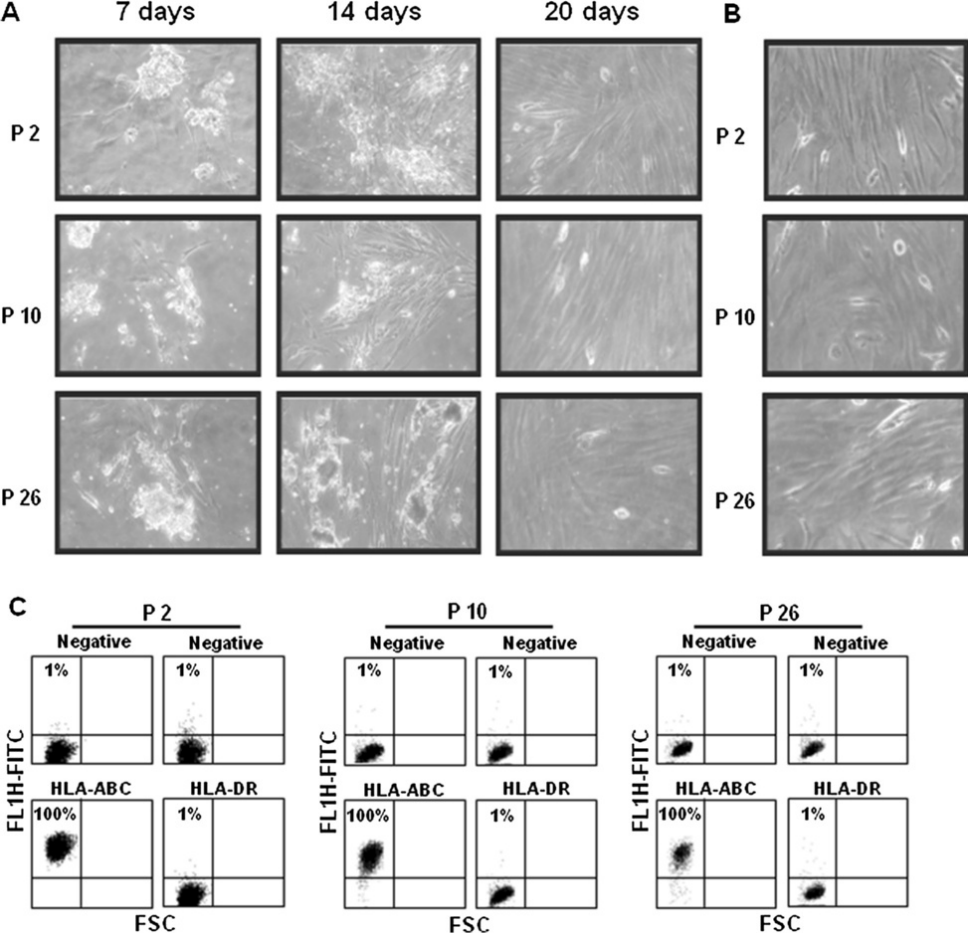
**MSCs obtained from human amniotic and chorionic membranes of term placentas. A)** Inverted light microscope images of cells derived from three different placentas (P2, P10, P26) at different days from adhesion on culture plastics. Original magnification: 40X. **B)** Inverted light microscope images of P2, P10 and P26 placenta adherent cells subcultured *in vitro*. The micrograph is an example of a five-day culture at 6 pd (passage 4). Original magnification: 40X. **C)** FCM analysis of HLA-ABC and HLA-DR antigens in P2, P10 and P26 placenta cultured cells. A negative sample was incubated with the corresponding isotype. The cytograms shown are representative of three different experiments with similar results. FCM, flow cytometry; MSCs, mesenchymal stem cells; pd, population doubling.

PD-MSCs showed plastic adherence and a typical fibroblastic-like morphology using light microscopy and developed visible colonies after seven days in 5% CO_2_ at 37°C. These colonies, in turn, started to proliferate steadily; the flasks were almost 60% to 70% confluent at day 14, and after 20 days in culture, the cell monolayer reached approximately 90% confluence (approximately 1.6 × 10^6^ cells/50 cm^2^) (Figure 1A). Therefore, the adherent cells readily propagated *in vitro* after successive cycles of trypsinisation; moreover, they formed subcultures that maintained the same fibroblast-like morphology and saturation density of those obtained after 20 days of culture (Figure 1B). The immunological properties of the PD-MSCs were analysed after 6 pd (corresponding to passage 4) of subculturing using FCM. These derived cells were strongly positive for the highly polymorphic HLA class I antigens (HLA-A, HLA-B, HLA-C), and they were negative for MCH class II (HLA-DR), decisively identifying their foetal origin (Figure 1C).

**Figure 2:**
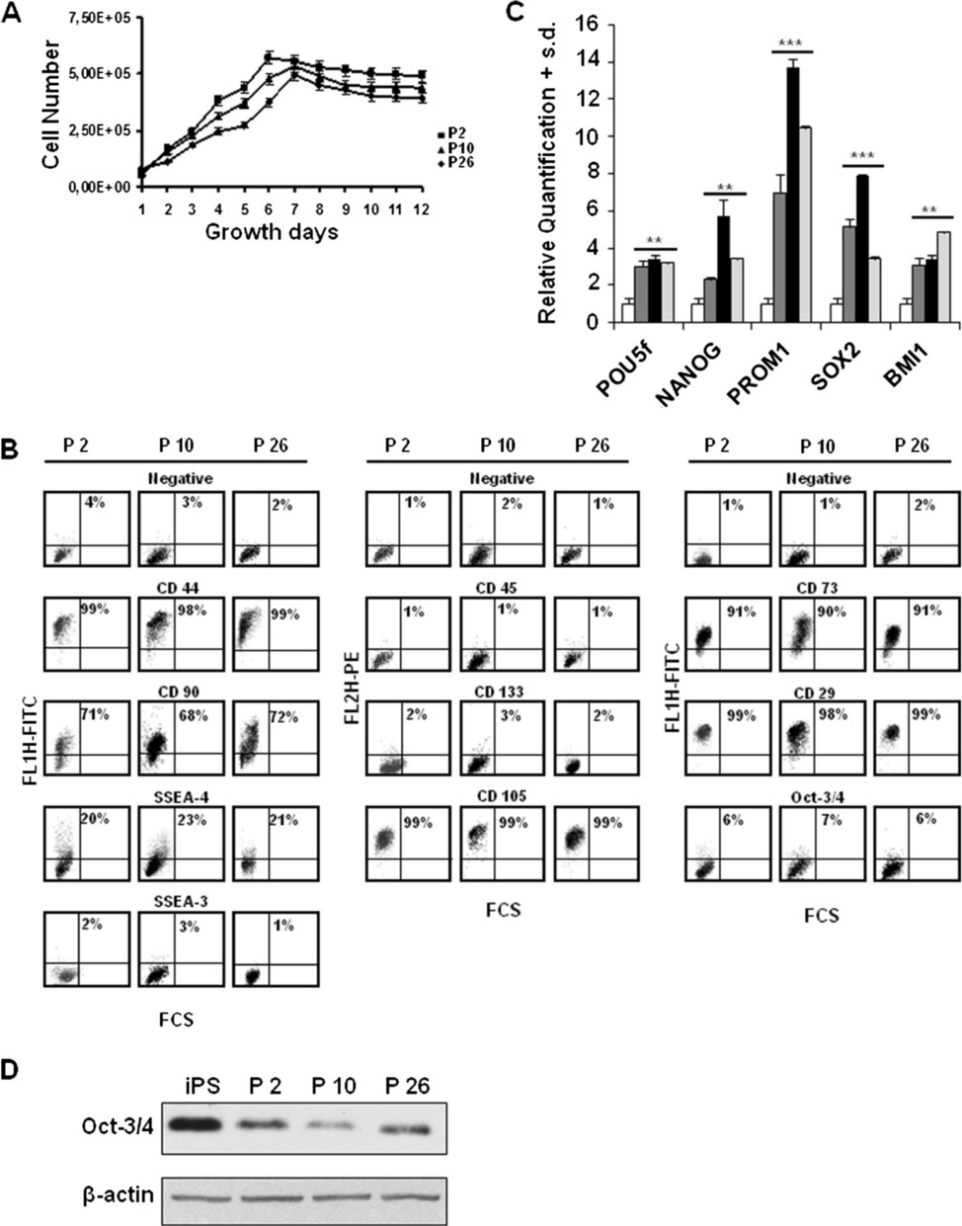
**Characterisation of PD-MSCs. A)***In vitro* growth curve of the PD-MSCs derived from P2, P10 and P26 placentas at 6 pd. **B)** FCM analysis of cell surface markers in five-day culture at 6 pd. A negative sample was incubated with the corresponding isotype. The cytograms relative to P2, P10 and P26 placentas are representative of three different experiments with similar results. **C)** Levels of the indicated mRNA in PD-MSCs from P2 (dark grey), P10 (black) and P26 (light grey) after five days of culture at 6 pd. The data are reported as the level of mRNA relative to the uncultured placenta and are the means + SD (n = 3). Statistical significance: **P <0.01; ***P <0.001. **D)** Levels of Oct-3/4 protein in PD-MSCs relative to P2, P10 and P26 placentas compared to iPS. Each lane was loaded with 25 μg proteins from cell lysates and β-actin was used as the control for loading equal amounts of proteins. The experiment was repeated three times showing similar results. Band intensities were measured by densitometry. FCM, flow cytometry; iPS, induced pluripotent stem cells; PD-MSCs, placenta-derived mesenchymal stem cells; pd, population doubling; SD, standard deviation.

Cells duplicated exponentially until day 7, when they reached a plateau phase; the mean doubling time of the exponential cell population was about 36 hours (Figure 2A). The percentage of cell viability was high (median value 93%, range 86% to 95%) (data not shown). The cell-surface phenotypes were characterised using FCM (Figure 2B). PD-MSCs were positive for MSC markers, such as CD44, CD73, CD90, CD29, and CD105, and for the stage-specific embryonic antigens SSEA-4, and they were negative for SSEA-3 and Oct -3/4. The haematopoietic marker CD45 and the antigen CD133 were not expressed.

On the other hand, PD-MSCs showed an increase in the expression of genes related to embryonic stem cell phenotypes, including *POU5f* (about 3-fold) and *NANOG* (with a maximum value of about 6-fold), of the CD133 encoding gene *PROM1* (with a maximum value of about 14-fold) and of the other two stemness-related genes, *SOX2* (with a maximum value of about 8-fold) and *BMI1* (about 4-fold), compared with non-cultured placentas (Figure 2C). To really demonstrate that PD-MSCs have a stemness signature we compared the expression of the main stemness-related genes (*POU5f*, *NANOG* and *SOX2*) in these cells with those in iPS cells used as positive control. The Δct values of the indicated genes in iPS cells, uncultured placenta and in three different cultured placentas evaluated by real time PCR are shown in Additional file 1: Figure S1. Δct values, calculated by the Δct method after normalising real time RT-PCR Ct on the average of the endogenous controls, are inversely correlated with the amount of the gene present in the sample. It is evident that the expression of the three genes appears upregulated in iPS cells and in PD-MSCs with respect to uncultured placenta, thus further demonstrating that the PD-MSCs have features of embryonic stem cells.

Considering the key role of the stemness-related factors, such as Oct-3/4 SSEA-3 and CD133, and not having a clear demonstration of their expression by FCM we performed western blot analysis of these proteins. We found the presence of Oct-3/4 in all three cultured placentas (Figure 2D), while for SSEA-3 and CD133 expression, the western blotting confirmed the absence of these molecules in PD-MSCs (data not shown).

The PD-MSCs displayed no visible changes in terms of their morphology, cell proliferation, phenotypic patterns or gene expression profiles at up to 29 pd (data not shown).

### PD-MSCs acquire a neuronal differentiated phenotype

**Figure 3:**
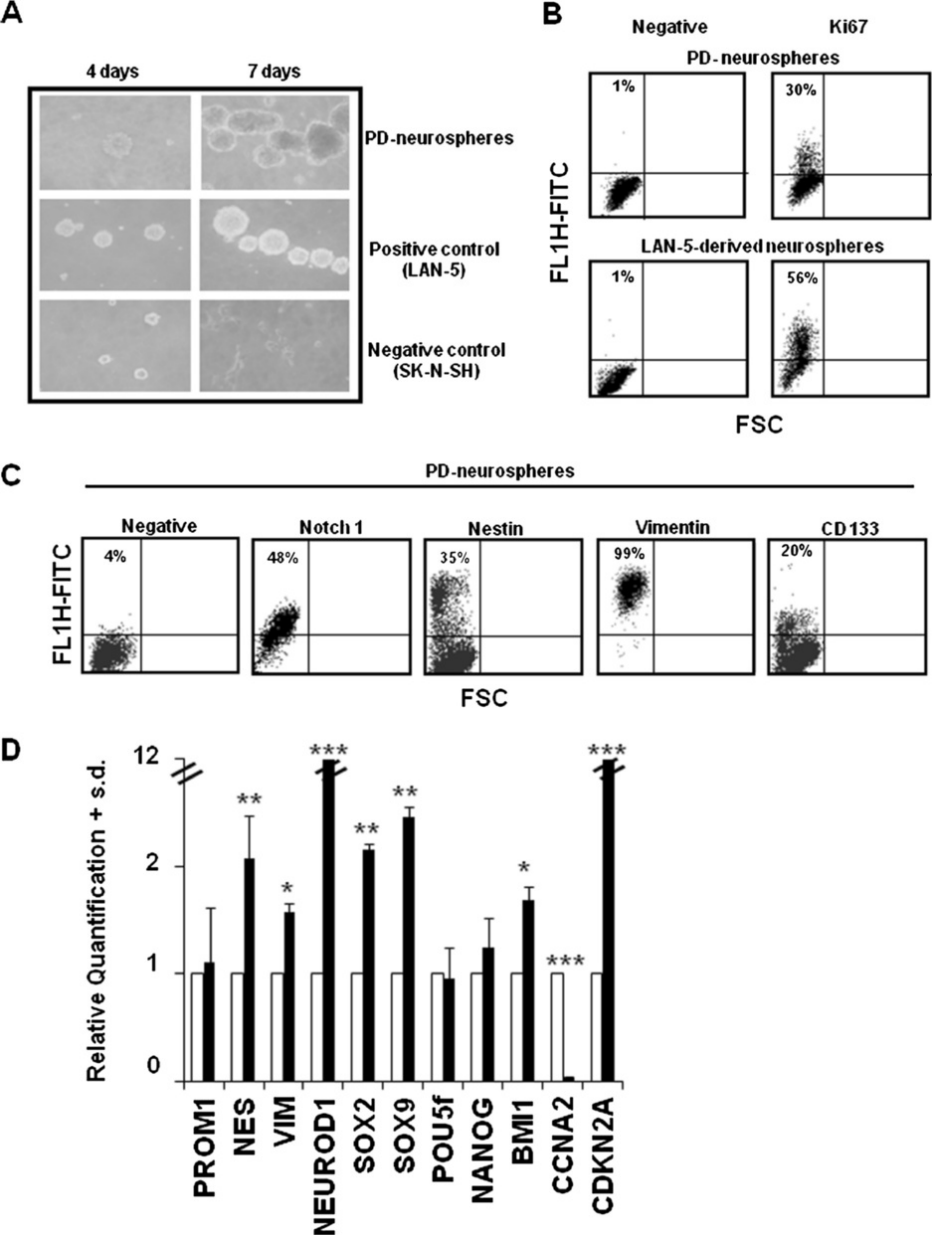
**Neural lineage induction and terminal differentiation. A)** Secondary PD-neurospheres after four and seven days of culture in the non-adhesion conditions. LAN-5 and SK-N-SH NB cells were used as positive and negative controls, respectively. **B)** FCM analysis of the cell cycle related marker Ki67 in secondary PD-neurospheres after seven days of culture. The negative is the sample incubated with the corresponding isotype. The cytograms shown are representative of three different experiments with similar results. **C)** FCM analysis of the indicated neural stem cells markers in secondary PD-neurospheres after seven days of culture. The negative is the sample incubated with the correspondent isotype. The cytograms shown are representative of three different experiments with similar results. **D)** Levels of the indicated mRNA in secondary PD-neurospheres (black) after seven days of culture. The data are reported as the level of mRNA relative to the PD-MSCs (white) after five days of culture at 3.6 pd (passage 4) and are the means + SD (n = 3). Statistical significance: *P <0.05; **P <0.01; ***P <0.001. FCM, flow cytometry; PD-MSCs, placenta derived-mesenchymal stem cells; pd, population doubling; SD, standard deviation.

Because it has been reported that amnion and chorion cells acquire an ectodermal-lineage phenotype *in vitro* and, in particular, are able to differentiate into neurons [18], we induced PD-MSCs into the neural lineage by sphere-forming assay using placenta P10. After four days, PD-MSCs formed primary floating neurosphere-like structures (data not shown). Secondary neurospheres were obtained after the disaggregation of primary neurosphere-like structures into single cells and subsequent culture under identical culture conditions. After seven days, the newly formed spheres grew rapidly in size, whereas non-proliferating cells died (Figure 3A). For a positive control, we used the LAN-5 NB cell line, which is known to form spheres in non-adherent culture conditions. To confirm that the increase in colony size was due to the proliferation, we performed Ki67 immunostaining on secondary dissociated spheres at day 7. We found that approximately 30% of the secondary PD-neurospheres population showed positive antigen expression, indicating that these spheres derive from cycling cells and not by re-aggregation of the dissociated cells (Figure 3B). As a control, we used neurospheres derived from the LAN-5 NB cell line. We then characterised the PD secondary neurospheres for the expression of some neural lineage-related markers. The spheres showed approximately 48% Notch 1, 35% nestin, 99% vimentin and 20% CD133 FCM positivity (Figure 3C). The gene expression profile of the PD secondary neurospheres showed that the *NES*, *VIM* and *NEUROD1* genes were up-regulated compared with their levels in PD-MSCs, whereas *PROM1* remained at an expression level similar to that in the PD-MSCs. The stemness-related genes *SOX2*, *SOX9* and *BMI1* were up-regulated as well, confirming the stem cell-like features of these neurospheres. By contrast, the expression of embryonic stem cell-related genes (*POU5f* and *NANOG*) did not significantly change when compared with PD-MSCs. Moreover, we also found a significant decrease of the expression of the gene encoding for cyclin A (*CCNA2*), which is involved in the control of S phase progression. These data are consistent with the increase of the cell-cycle cdks inhibitor gene *CDKN2A* encoding for p16INK4 (Figure 3D).

**Figure 4:**
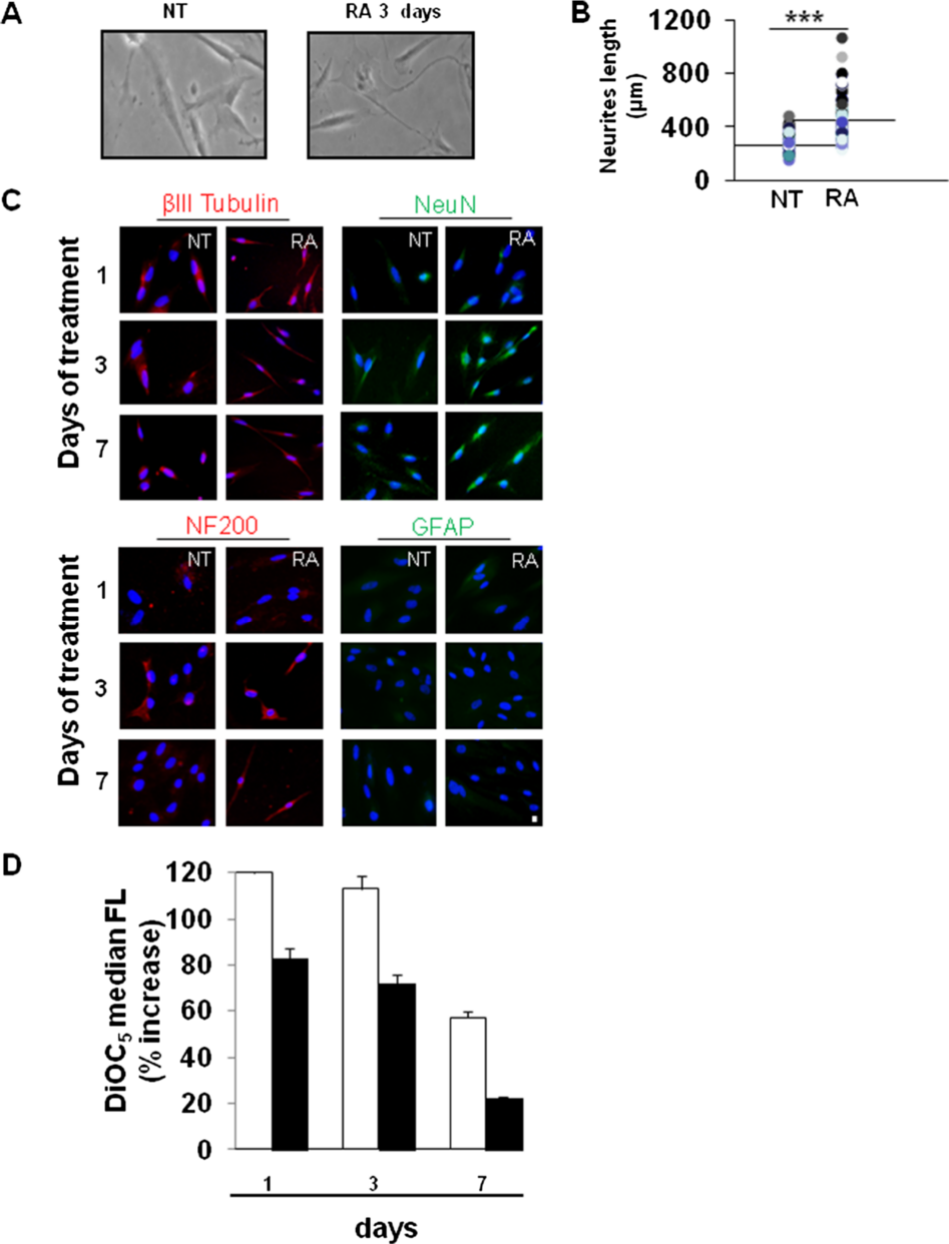
**Adherent PD-neurospheres differentiate upon RA. A)** Inverted light microscopic images of adherent PD-neurospheres (NT) or RA-treated for three days. Original magnification: 40X. The images shown are representative of three different experiments with similar results. **B)** Neurites length (μm) analysis. Distribution of the neurite outgrowth length as measured in adherent PD-neurospheres treated with RA for three days. Statistical significance: ***P ≤0.001. **C)** Representative fluorescent images of PD-neurospheres expanded for 12 passages and exposed to RA for one, three and seven days. Red = NF200 and βIII tubulin, Green = GFAP and NeuN immunostaining and blue = HOECHST. Scale bar: 10 microns. **D)** Distribution of intracellular DiOC_5_ fluorescence in adherent PD-neurospheres treated or not with RA for one, three and seven days expressed as % increase of treated cells versus untreated (black). Valinomycin treated cells (white) were used as hyperpolarised control. The result shown is representative of three different experiments overlapping. GFAP, glial fibrillary acidic protein; PD, placenta-derived; RA, retinoic acid.

To verify the ability of the PD-neurospheres to differentiate into neurons, we subcultured the dissociated spheres in a medium containing serum and we investigated the morphological and functional changes that occurred in adherent PD-neurospheres after RA. In Figure 4A we show light micrographs of cells grown with or without RA indicating that untreated cells had a fibroblast-like morphology; but upon exposure to RA, the cells changed to a neuron-like shape with clear evidence of the presence of peripheral projection from the cell body strongly remindful of neurites outgrowth. The number of cells with neurites increased approximately 90% after RA treatment. As shown in Figure 4B the neurite length evaluated in RA treated cells had a median value of 453 μm (range = 261 to 1067) compared to untreated cells with a median value of 282 μm (range = 190 to 436). Moreover, the expression of early- or late-stage neuron markers such as βIII-tubulin, neurofilament 200 (NF200) and neuronal nuclei (NeuN) after RA exposure demonstrated the ability of such cells to terminally differentiate into neurons. On the other hand, the same differentiation protocol did not reveal the presence of GFAP-positive astrocytes (Figure 4C). In order to evaluate whether differentiated adherent PD-neurospheres showed a neuron function, we measured membrane hyperpolarization which is typical of differentiated neurons by using the fluorescent membrane potential probe DiOC_5_. As shown in Figure 4D we found a hyperpolarization of the plasmatic membrane in RA treated cells evident already at day 1. The hyperpolarization found in these cells was comparable to that obtained in valinomycin treated cells used as positive control.

Similar results were obtained also using P2 and P26 (data not shown).

### PD-neurospheres spontaneously transform

**Figure 5:**
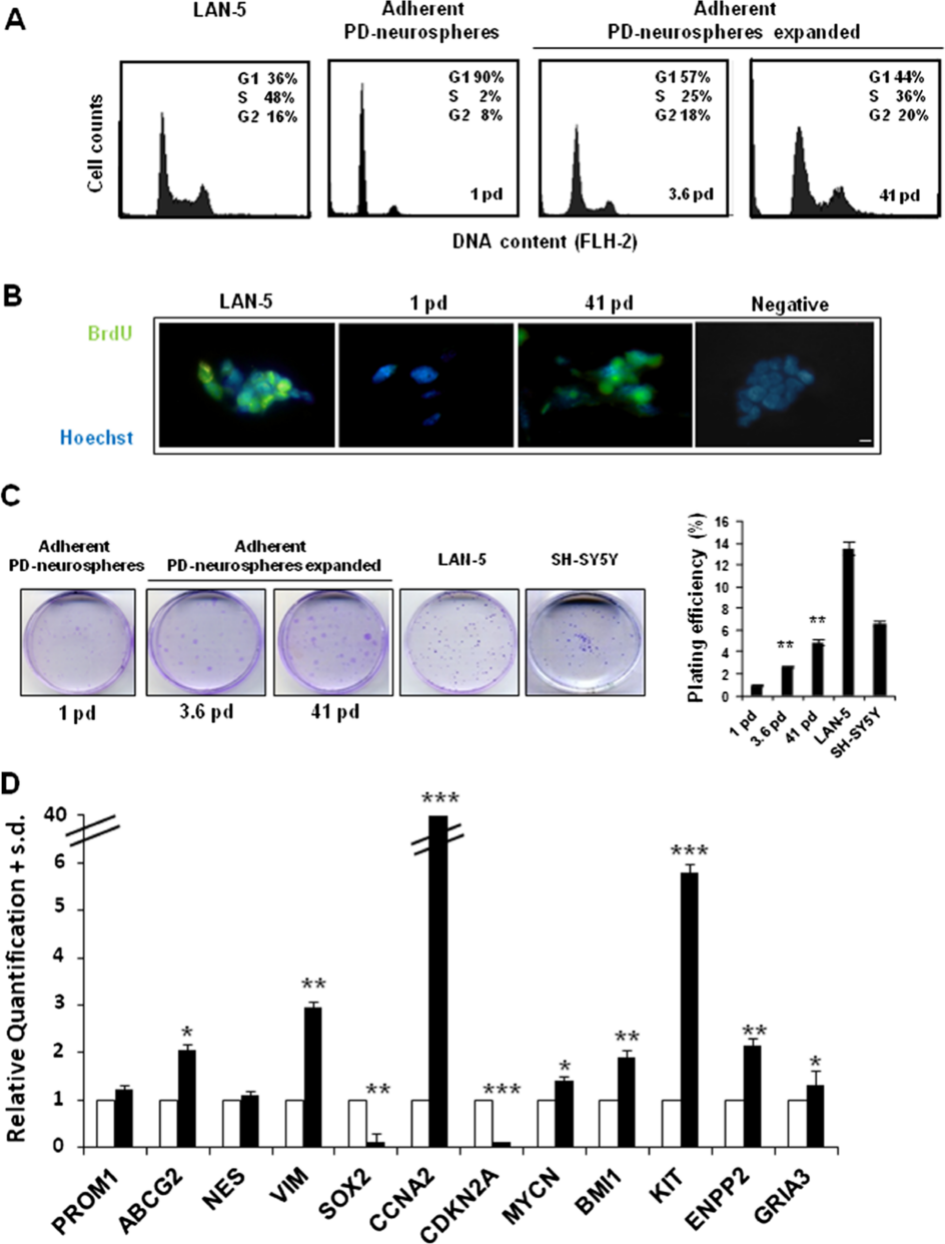
**Propagation and spontaneous transformation of secondary PD-neurospheres. A)** Cell cycle FCM analysis in PD-neurospheres expanded for 3.6 and 41 pd (3 and 19 passages, respectively). The unexpanded secondary PD-neurospheres are also shown (1 pd, 0 passages). As a control of cell proliferation of an established cancer cell line we used LAN-5 cells. **B)** Representative fluorescent images of PD-neurospheres expanded for 41 pd. Green, BrdU immunostaining and blue, HOECHST. Scale bar: 20 microns. **C)** Left, images of the *in vitro* colonies obtained in secondary PD-neurospheres (1 pd) and in PD-neurospheres at different passages of expansion (3.6 and 41 pd) seeded at clonal density and evaluated after 15 days of culture. Right, plating efficiency values (+SD; n = 5) in the same samples. As positive control we used the LAN-5 and SH-SY5Y cell lines. Statistical significance: **P <0.01. **D)** Levels of the indicated mRNA in PD-neurospheres expanded for 41 pd (black). The data are reported as the level of mRNA relative to the secondary PD-neurospheres (white) and are the means + SD (n = 3). Statistical significance: *P <0.05; **P <0.01; ***P <0.001. BrdU, bromodeoxyuridine; FCM, flow cytometry; PD, placenta-derived; pd, population doubling; SD, standard deviation.

PD-neurospheres were expanded as adherent cultures for several population doublings. At 3.6 pd these cells already showed an increase in the percentage of S-phase cells (%S = 25) compared with uncultured neurospheres (%S = 2). We monitored cell proliferation at different population doublings; at 41 pd, the percentage of S phase cells was maintained at high levels (%S = 36). As a control, we used the *in vitro* established LAN-5 NB cell line (Figure 5A). To confirm the proliferation activity, we measured BrdU incorporation into adherent PD-neurospheres in both unexpanded cells (1 pd) and after 41 population doublings (41 pd). A strong BrdU incorporation was evident at 41 pd compared with unexpanded neurospheres, confirming that the expanded neurospheres maintained a proliferative potential *in vitro*. LAN-5 NB cell BrdU incorporation was used as a control (Figure 5B).

The colony formation assay also demonstrated that these cells retained proliferative potential after *in vitro* expansion. Plating efficiency, evaluated in the adherent PD-neurospheres after 15 days of *in vitro* culture, was increased by approximately five-fold during the expansion, achieving values comparable to those observed in established NB cell lines, such as LAN-5 and SH-SY5Y (Figure 5C). In addition, calculation of the mean doubling time of the adherent PD-neurospheres expanded cell population was 26 hours (data not shown). These results further demonstrate the transforming capacity of these cells.

The ability of adherent PD-neurospheres to transform during their *in vitro* expansion was also demonstrated by analysing the expression of some related genes using qRT-PCR. Figure 5D shows that changes in gene expression levels were related to stemness, proliferation and tumour transformation. Interestingly, the expression of the gene *CCNA2* was increased by approximately 40-fold and *CDKN2A* was decreased by approximately 90-fold, confirming the substantial increase in proliferative potential that is a characteristic of transformed cells. Moreover, the expression levels of genes that are related to a more aggressive phenotype in NB cells (for example, *ENPP2*, *GRIA3*) were increased by approximately two-fold. Furthermore, we found increased expression levels of the *MYCN* gene (approximately 1.5-fold), which is a hallmark of NB.

Interestingly, cytogenetic analysis using the Array-Based Comparative Genomic Hybridization (aCGH) assay did not reveal any pathogenetic alteration (data not shown).

**Figure 6:**
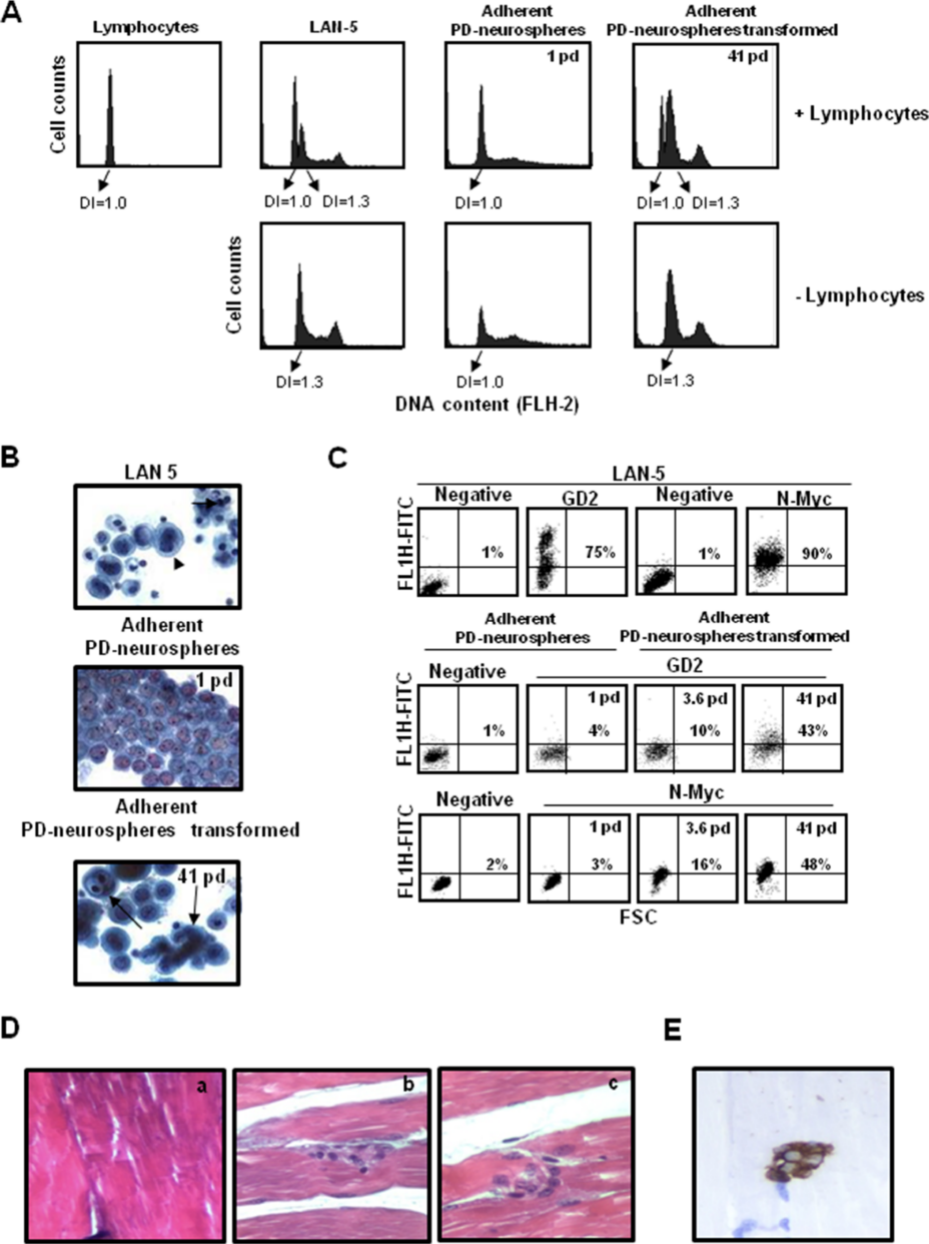
**Characterisation of the neoplastic phenotype. A)** Frequency histograms of cellular DNA content in PD-neurospheres unexpanded and expanded for 41 pd. Lymphocytes were used as normal diploid internal standard. LAN-5 NB cells were used as aneuploid DNA content. Ploidy is expressed as DNA index (DI), which represents the ratio of relative G0/G1 DNA content of sample versus normal diploid lymphocyte. **B)** Papanicolau staining of PD-neurospheres unexpanded and expanded for 41 pd. LAN-5 NB cells were used as tumour control. The arrows indicate multinucleated abnormal cells with large prominent nucleoli. Original magnification: 40X. **C)** GD2 and N-Myc expression in secondary unexpanded PD-neurospheres (1 pd) and in PD-neurospheres expanded for different passages (3.6 pd and 41 pd). The negative sample was incubated with the corresponding isotype. The LAN-5 NB cells were used as a positive control. The cytograms shown are representative of three different experiments with similar results. **D)** Spontaneous malignant transformation of adherent PD-neurospheres expanded injected subcutaneously into the flanks of NOD.CB17-Prkdc^scid^/NCrHsd mice. Haematoxylin and eosin staining of tissue sections fixed in 4% formalin and paraffin embedding confirmed the infiltrative tumour phenotype. Original magnification: 40X. **a)** healthy muscle tissue; **b** and **c)** neoplastic nests of cells in muscle tissue. **E)** HLA-ABC immunohistochemistry reactivity of tissue sections fixed in 4% formalin and paraffin embedding confirmed the human origin. Original magnification: 40×. NB, neuroblastoma; PD, placenta-derived; pd, population doubling.

Since abnormal ploidy is a cellular feature frequently associated with solid tumours we studied the DNA content by FCM. Flow cytometric DNA content measurement of adherent PD-neurospheres unexpanded (1 pd) displayed a diploid DNA content (DI = 1.0) indistinguishable from normal lymphocytes. By contrast, adherent PD-neurospheres transformed *in vitro* for 41 pd showed an abnormal hyperdiploid DNA content with a DI value of 1.3. The LAN-5 cells have a DI value of 1.3 (Figure 6A). The cytological analysis of the same samples demonstrated the presence of multinucleated abnormal cells with large prominent nucleoli and increased nucleo/cytoplasm ratio in PD-neurospheres transformed (41 pd); whereas the unexpanded adherent neurospheres (1 pd) showed a normal morphology (Figure 6B). These data further demonstrate that the adherent PD-neurospheres during the *in vitro* expansion progress towards a cancer phenotype. The transformation of adherent PD-neurospheres versus an NB tumour phenotype was demonstrated by the presence of the NB-specific cell surface marker, disialoganglioside GD2 and the N-Myc protein (Figure 6C).

In addition, since the acquisition of mesenchymal markers could be associated with cancer invasion we also analysed the expression of mesenchymal markers in the transformed cells. Our cells maintained the expression of CD29, CD90, CD73, CD105 and CD44 at a similar extent to PD-MSCs (see Additional file 2: Figure S2).

To definitely demonstrate the transformation of adherent PD-neurospheres after expansion *in vitro*, we evaluated the tumourigenic potential of these cells by injecting them in NOD.CB17-Prkdc^scid^/NCrHsd mice. Even though no visible tumour mass was evident, after five months mice were sacrificed to perform histological analysis of the tissues from the injected sites. Histopathological examination demonstrated the presence in normal muscle tissue of large cells with an infiltrative tumour phenotype showing vesicular nuclei and prominent nucleoli and arranged in neoplastic nests (Figure 6D). We confirmed that the engrafted neoplastic cells in the NOD.CB17-Prkdc^scid^/NCrHsd mice originated from a human source using an anti-HLA class 1 ABC monoclonal antibody by the immunoperoxidase staining method (Figure 6E).

Similar results were obtained also using P2 and P26 (data not shown).

## Discussion

The CSC model links neoplastic cells with normal stem cell biology. Normal stem cells may be functionally defined as cells with the capacities of self-renewal [19], multipotency [10] and differentiation [18]. This hierarchical stem cell model is attractive because it provides a likely explanation for multiple treatment failures and ideal cellular targets to definitively eradicate refractory tumours [20]. To date, stem cell–like cells have been convincingly identified in a number of tumour types, including blood, breast, brain, prostate, skin, colon and lung cancers [4-9]. We suggest the possibility that such a model may not apply to all solid tumours or may turn out to be considerably more complex in some cancers. Regarding NB, whether it results from the CSC model or from clonal evolution within tumours is still a matter of intense debate. Recently, Coulon and colleagues [3] have described an approach to identify and characterise CSCs in a panel of NB tumours, suggesting that the tumours’ embryonic origin and heterogeneity qualifies NB tumours as a pertinent model to explore CSC models in solid tumours. Thus, other authors [11,12] isolated highly tumourigenic, drug-resistant, sphere-forming cell lines from NB metastatic bone marrow even though no specific gene expression profiles have been identified for NB CSCs, either in primary tumours or in NB cell lines [3]. Moreover, because of their plasticity and the influence of the environment, it is still unclear to what extent the existence of an NB-CSC ‘niche’ is dependent on the presence of the NB-CSCs themselves. Indeed, the phenotypic identity of the NB-CSCs may shift as the niche changes, and *vice versa*, making the identification of NB-CSCs even more difficult. Thus, the development of stem cell models to study the molecular mechanisms by which neoplastic transformation occurs could be useful. The biological features and the absence of ethical issues concerning the use of stem cells isolated from human membranes from term placentas has allowed us to identify a model to study spontaneous stem cell transformation. Here, we report that normal human stem cells from amniotic and chorionic placenta membranes are forced into a neural lineage, expanded in long-term cultures and immortalised by acquiring an NB-like phenotype. Placenta has attracted increasing attention over the past decade as a stem cell source [21]. Consistent with Bacenkova and colleagues [22], our data show that amnion and chorion PD stem cells display plastic adherence and fibroblast-like growth. Moreover, these PD cells were found to be of foetal origin. The use of foetal stem cells to study spontaneous transformation, compared with adult stem cells, appears to be advantageous due to the foetal stem cells’ expansion capacity and accessibility [23]. To assess the stem cell-like properties of amnion and chorion PD cells, the expression of stem cell markers and differentiation capacity have been examined. These cells expressed typical mesenchymal markers (CD44, CD73, CD90, CD29, CD105) but not haematopoietic markers, such as CD45, or neural progenitor markers, such as CD133 [18], even though we showed that the CD133 encoding gene PROM1 is expressed. Moreover, our cells expressed markers associated with pluripotent (POU5f, NANOG, SOX2 and BMI1) and multipotent (SSEA-4) human embryonic stem cells, as also reported by Bacenkova and colleagues [22]. However, in contrast to these authors, we did not detect the expression of SSEA-3 protein. This contradictory finding may be due to the different techniques used to detect this antigen (we used FCM, whereas they used immunocytochemistry). On the other hand, Ilancheran and colleagues [18] reported that SSEA-3 expression has yet to be confirmed in MSCs obtained from foetal membranes.

According to different authors [24-26], we neuralised MSCs derived from amnion and chorion membranes, obtaining a model able to acquire neuronal phenotypic and functional characteristics under specific conditions.

Recent publications present conflicting evidence about the transformation propensity of human MSCs. Whereas Gro Vatne Røsland and collaborators [15] observed spontaneous malignant transformation of bone marrow-derived adult MSCs *in vitro*, other investigators have reported that MSCs remain stable with no evidence of transformation in long-term cultures of both bone marrow and adipose tissue-derived human MSCs [27,28]. For the first time, we show that adherent PD-neurospheres after long-term *in vitro* expansion were able to spontaneously transform, showing an aneuploid DNA content even with a CGH assay negative for the presence of pathogenic copy number variations. It is likely that this technology, even with high sensitivity and resolution compared to conventional GTG-binding, may not be sufficient to detect a low proportion of cells with abnormalities and balanced rearrangements suggesting that changes in the expression of genes, and not in their amplification, occurred in our cells. These cells were highly proliferative and formed colonies *in vitro* with cloning efficiency values similar to those obtained in NB cell lines. These data are consistent with an increase in *CCNA2* gene expression associated with a decrease of *CDKN2A*, indicating that an alteration of the pathway involved in cell cycle control may efficiently extend the proliferative lifespan, thus predisposing adherent PD-neurospheres to neoplastic transformation. In agreement with Coulon and colleagues [3], we found an up-regulation of genes described as CSC markers, such as *PROM1*, *ABCG2*, *NES* and of genes involved in the reprogramming such as *BMI1*[29].

Moreover, we also found an over-expression of *MYCN*, *ENPP2* and *GRIA3* genes previously shown to play a key role in the development and aggressiveness of the NB process [30]. Following Rubio *et al*., we hypothesize that PD-neurospheres malignant transformation occurred bypassing senescence by upregulating *MYCN* and repressing *CDKN2A* genes. Interestingly, we observed a reduction of the *SOX2* gene expression levels, thus confirming the neuronal fate. Agreeing with Cohen and collaborators [31], we found that an upregulation of the *KIT* gene played a significant role in NB growth regulation even if its role in neuronal differentiation is less clear.

Moreover, we demonstrated the transformation of adherent PD-neurospheres after expansion *in vitro* by injecting them in NOD.CB17-Prkdc^scid^/NCrHsd mice. Interestingly, we found the presence of MSC markers in our PD-neurospheres transformed. Our hypothesis is that, despite the existing difficulty of obtaining tumour formation after injection of MSCs into mice, the MSC features could contribute to the uptake of the tumour in mice.

## Conclusions

In this study, we developed a CSC model starting from normal human stem cells derived from amniotic and chorionic placenta membranes. We demonstrated that, under specific differentiation conditions, human amnion and chorion membrane-derived MSCs can be converted into neural lineages. In addition, after extensive culture, *in vitro*, these cells undergo spontaneous transformations and acquire an NB-like phenotype.

## Abbreviations

BrdU: Bromodeoxyuridine; CSCs: Cancer stem cells; DI: DNA index; (D)MEM: (Dulbecco’s) modified Eagle’s medium; FCM: Flow cytometry; FCS: Foetal calf serum; FITC: Fluorescein isothiocyanate; GD2: Disialoganglioside; GFAP: Glial fibrillary acidic protein; HLA-ABC: Human highly polymorphic class I leukocyte antigens; HLA-DR: Human class II leukocyte antigen; iPS: Induced pluripotent stem cells; MCH: Major histocompatibility complex; MSCs: Mesenchymal stem cells; NB: Neuroblastoma; NF200: Neurofilament 200; PBS: Phosphate-buffered saline; PD: Placenta-derived; pd: Population doubling; PI: Propidium iodide; RIN: RNA integrity number; SSEA: Stage-specific embryonic antigen; TICs: Tumour-initiating cells.

## Competing interests

The authors declare that they have no competing interests.

## Authors’ contributions

DA: involved in drafting the manuscript, development of methodology, acquisition of data, analysis and interpretation of data. MN: involved in drafting the manuscript, acquisition of data, analysis and interpretation of data. LG: involved in drafting the manuscript, acquisition of data, analysis and interpretation of data. LC: involved in drafting the manuscript, acquisition of data. EC: involved in drafting the manuscript, acquisition of data. VA: involved in drafting the manuscript, acquisition of data. MP: involved in drafting the manuscript, acquisition of data. CL: involved in drafting the manuscript, acquisition of data. CB: involved in drafting the manuscript, analysis and interpretation of data. RR: involved in drafting the manuscript, analysis and interpretation of data. IDA: conception and design, analysis and interpretation of data, writing, review and/or revision of the manuscript. AS: involved in drafting the manuscript, analysis and interpretation of data. GN: involved in drafting the manuscript, analysis and interpretation of data. BB: conception and design, analysis and interpretation of data, writing, review and/or revision of the manuscript. All authors read and approved the final manuscript.

## Supplementary Material

Additional file 1: Figure S1 Expression of the main stemness-related genes in PD-MSCs compared with iPS positive control. The indicated mRNA levels were measured, by real time RT-PCR determining Δct values normalised against the average of the endogenous controls, in iPS (stacked lines), uncultured placenta (white) and three different cultured placentas, P2 (grey), P10 (black) and P26 (light grey). Δct values, calculated by the Δct method after normalizing real time RT-PCR Ct are inversely correlated with the amount of the gene present in the sample. The experiment was repeated three times with superimposable results. Click here for file

Additional file 2: Figure S2 FCM analysis of mesenchymal markers in adherent PD-neurospheres transformed at 41 pd. Negative is the sample incubated with the corresponding isotype. The cytograms are representative of three different experiments with similar results. Click here for file
