# Supplementary figures and images for: Human placenta-derived neurospheres are susceptible to transformation after extensive in vitro expansion
Source: Stem Cell Res Ther. 2014 Apr 22;5(2):55. doi: 10.1186/scrt444 (PMC4055136; doi:10.1186/scrt444)

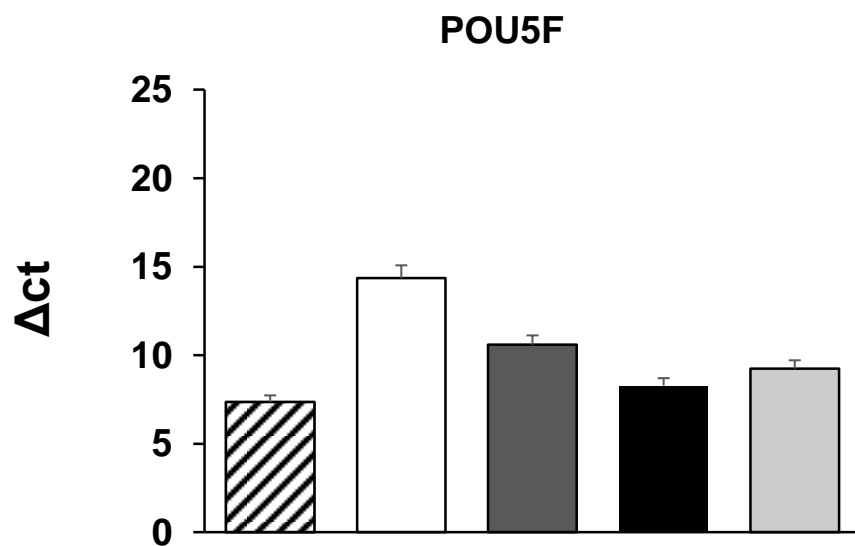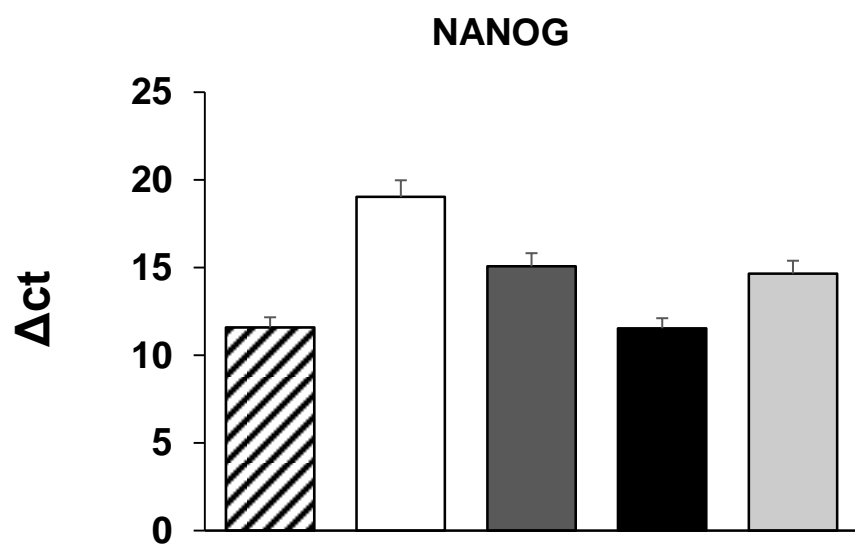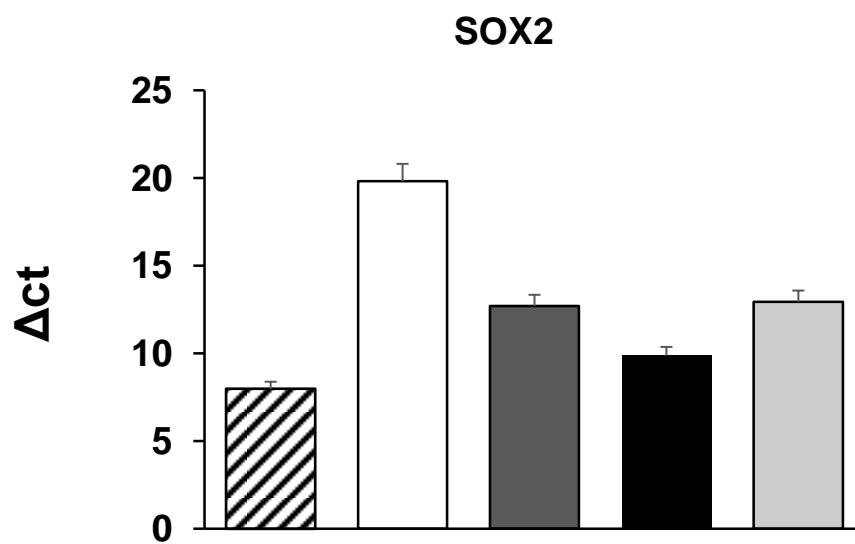

**Figure S1**

Supplement: Additional file 1: Figure S1 — Expression of the main stemness-related genes in PD-MSCs compared with iPS positive control. The indicated mRNA levels were measured, by real time RT-PCR determining Δct values normalised against the average of the endogenous controls, in iPS (stacked lines), uncultured placenta (white) and three different cultured placentas, P2 (grey), P10 (black) and P26 (light grey). Δct values, calculated by the Δct method after normalizing real time RT-PCR Ct are inversely correlated with the amount of the gene present in the sample. The experiment was repeated three times with superimposable results. [file scrt444-S1.pdf]

### Adherent PD-neurospheres transformed

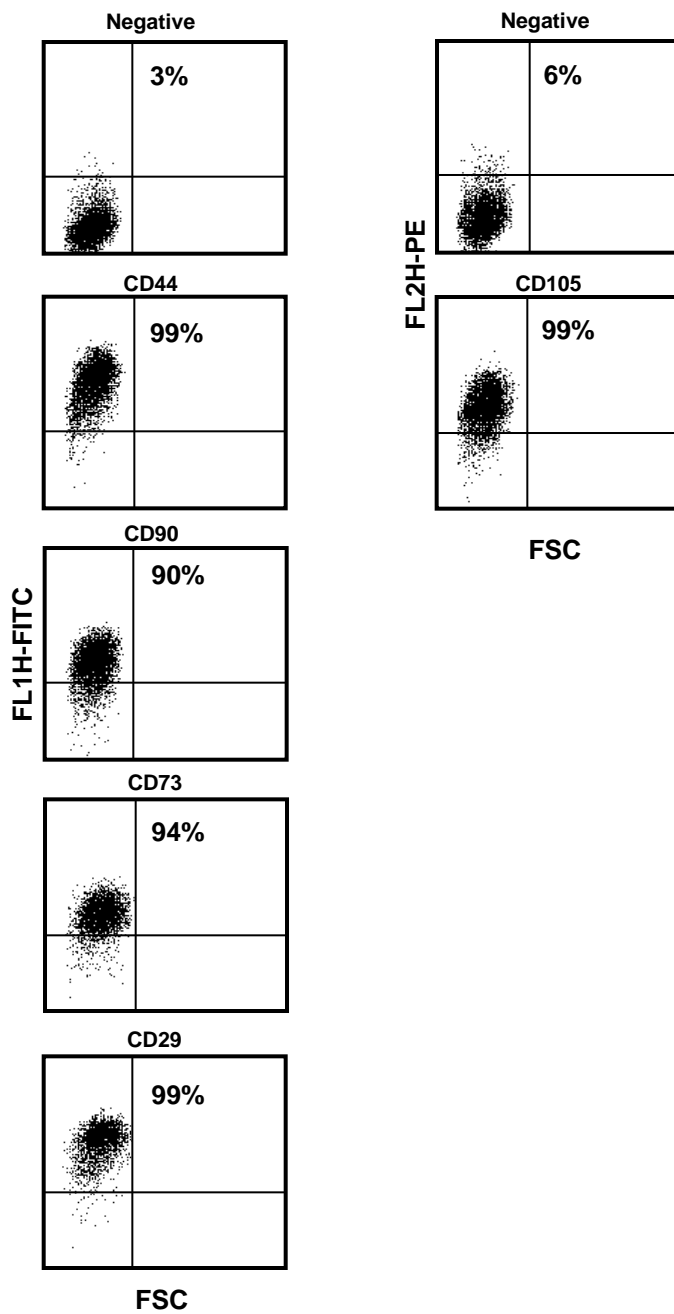

**Figure S2**

Supplement: Additional file 2: Figure S2 — FCM analysis of mesenchymal markers in adherent PD-neurospheres transformed at 41 pd. Negative is the sample incubated with the corresponding isotype. The cytograms are representative of three different experiments with similar results. [file scrt444-S2.pdf]
